# Deer Antler Uridine Regulates Glycolysis in Microglia via HSP90/HIF‐1α to Improve Cognitive Impairment in Alzheimer's Disease Mice

**DOI:** 10.1111/cns.70416

**Published:** 2025-05-14

**Authors:** Yongjian Liu, Chenyang Han, Li Guo, Wenyan Li, Shasha Wu, Jian Sheng, Liping Zhai, Heping Shen

**Affiliations:** ^1^ Interventional Department First Affiliated Hospital of Dalian Medical University Dalian China; ^2^ The Second Affiliated Hospital of Jiaxing University Jiaxing Zhejiang China

**Keywords:** Alzheimer's disease, cognitive impairment, glycolysis, microglia, uridine

## Abstract

**Aim:**

To investigate the role and mechanism of uridine (URI), an active component in deer antler, in improving cognitive impairment in Alzheimer's disease (AD) mice.

**Method:**

The APP/PS1 mouse model was used for AD. After URI gavage administration, cognitive behavioral changes in mice were detected using the Morris water maze, eight‐arm maze, and novel object recognition tests. Levels of inflammatory cytokines and lactate, pyruvate in the cortex were measured. The proportions of IBA‐1 and CD86 cells in tissues were detected, and the expression of key glycolysis proteins was examined. Network pharmacology was employed to analyze the targets of URI‐AD‐glycolysis. AAV‐CMV‐shHSP90 was injected to knock down brain HSP90 levels to further explore the anti‐AD mechanism of URI. In vitro, primary microglia were used to detect the proportion of CD86+ M1 cells and glycolysis levels.

**Result:**

URI can improve cognitive impairment in AD mice, with significant changes in cognitive ability and behavior. URI reduces glycolysis levels, the proportion of M1 cells (CD86+), and the activation degree of microglia, while inhibiting the activation of HSP90‐HIF‐1α. Network pharmacology analysis revealed that HSP90 is a major target of URI. When HSP90 is inhibited, the effect of URI is diminished. In vitro experiments showed that URI can inhibit the M1 polarization of microglia and reduce glycolysis levels.

**Conclusion:**

URI can inhibit microglial glycolysis and M1 polarization via HSP90/HIF‐1α, thereby improving cognitive behavioral deficits in AD mice due to neuroinflammation. Uridine in deer antler is a novel small molecule for anti‐AD.

## Graphical abstract

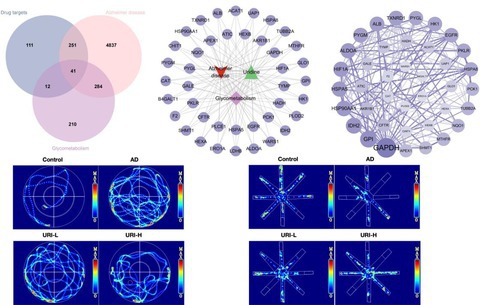

## 1 Background

Research progress on deer antler in the treatment of Alzheimer's disease (AD) has mainly focused on the mechanism of action of its active component, deer antler polypeptide (VAP). Multiple VAPs have been found to significantly improve AD through diverse mechanisms [1, 2, 3]. However, other active substances in deer antler have been relatively less studied. Uridine, a nucleoside compound extracted from the traditional Chinese medicine deer antler, has garnered attention in recent years due to its potential pharmacological effects [4]. Pharmacological studies in the nervous system have shown that uridine can intervene in metabolic processes within the body, promote the regeneration and repair of somatic cells, and thus maintain overall health [5, 6]. It can also reduce the levels of pro‐inflammatory cytokines such as TNF‐α, thereby alleviating inflammatory responses. The small molecule uridine can cross the blood–brain barrier to enter the brain region, regulating memory and neuronal plasticity in the central nervous system, and may possess neuroprotective effects. However, further research is needed to confirm this [7].

The polarization state of microglia is closely related to neuroinflammation in AD. M1‐type microglia are pro‐inflammatory cells that release a series of pro‐inflammatory mediators such as interleukin‐1β (IL‐1β), leading to neuronal apoptosis and potentially exacerbating inflammatory damage [8, 9, 10]. Energy metabolism is significantly associated with M1 polarization. Glycolysis is the primary energy metabolic pathway that promotes M1 polarization in microglia, with HIF‐1α being a key effector molecule regulating metabolic reprogramming and functional phenotypes of M1/M2 microglia [11]. After constructing HIF‐1α fragment transfection into microglia to induce overexpression of HIF‐1α, it was found that the expression levels of glycolysis‐related enzymes increased, along with the release of inflammatory cytokines. There are numerous upstream signals regulating HIF‐1α [12]. We mainly focused on glycolysis to investigate and elucidate the anti‐AD effects and mechanisms of uridine, providing new insights and references for the anti‐AD material basis of deer antler.

## 2 Materials and Methods

### 2.1 Mouse Grouping

We selected male C57BL/6J mice and APP/PS1 double transgenic AD mice models, purchased from Beijing Huafukang Biotechnology Co. Ltd. C57BL/6J mice served as wild‐type healthy controls, weighing 21–25 g, whereas the AD mice, aged 7 months and already exhibiting significant AD‐like lesions, also weighed 21–25 g. The mice were housed in an SPF environment with free access to food and water and consistent lighting.

Experiment 1: C57BL/6J mice were defined as the control group (healthy controls). AD mice constituted the model group. The URI group consisted of AD mice treated with URI at doses of 10 mg/kg and 20 mg/kg, respectively, administered daily by gavage, designated as URI‐L and URI‐H. URI was dissolved in distilled water, while the control and AD groups received equivalent volumes of distilled water daily by gavage.

Experiment 2: C57BL/6J mice were defined as the WT group. AD mice received AAV‐CMV‐shHSP90 (Vigene Biosciences, Shandong, China) via intracranial stereotactic injection at a dose of 9 × 10^13^vg/mL, 100 nL, at a rate of 10 nL/min, designated as AD‐KO. Additionally, in the AD‐KO group, URI was administered at 20 mg/kg daily by gavage, designated as AD‐KO + URI. The remaining three groups received equivalent volumes of distilled water daily by gavage.

### 2.2 Morris Water Maze [13]

Food‐grade titanium dioxide was used to bleach the experimental water, with the water temperature maintained at 22°C–25°C. The platform was placed in the center of the pool, 1 cm below the water surface. Before the experiment began, mice were familiarized with the swimming environment. Each mouse was placed in the water to adapt for 1–3 min and then allowed to stay on the platform for 20 s. For the formal directional navigation experiment, mice were placed in the pool from four quadrants and swam for 60 s to locate the platform. Mice that failed to find the platform within 60 s were guided onto it. This training lasted for 4 days. After the directional navigation experiment, the platform was removed, and mice were placed in the pool to observe their swimming routes and the number of times they crossed the platform, assessing spatial memory.

### 2.3 Eight‐Arm Maze [14]

The eight‐arm maze consists of eight identical arms radiating from a central point, with sensors at the beginning and end of each arm and food (bait) slots at the ends. Mice were food‐deprived for 12 h before each daily experiment and trained for 5 days, twice daily. The maze location, lighting, and temperature were kept consistent daily. On test day, food was placed in arms 2, 4, 6, and 8. Mice were placed in the center of the maze, allowed to adapt for 15 s, and then released to forage for 5 min. On the sixth day, the mice’ foraging behavior was observed for 5 min, recording the number of reference memory errors (RME, entering arms without food), working memory errors (WME, re‐entering a food arm), and the total time to consume all food. If the rat did not finish the food within 5 min, it was recorded as 5 min.

### 2.4 Novel Object Recognition Test [15]

The novel object recognition test consists of three stages. On day 1, no objects were placed in the environment, allowing mice to move freely and adapt to the surroundings. On day 2, two objects of the same shape but different colors (Object A) were placed in the environment, and mice were allowed to explore for 5 min. On day 3, one object was replaced with another of the same color but different shape (Object B). The time and frequency of mice exploring the new object B were recorded, analyzing the proportion of time and frequency of exploring Object B relative to the total time and frequency of exploring both objects.

### 2.5 ELISA

Levels of inflammatory cytokines IL‐6, IL‐1β, and TNF‐α in the mouse cortex were measured. In vitro experiments measured these cytokines in the culture supernatant after centrifugation. ELISA kits were purchased from Nanjing Jiancheng Bioengineering Institute. Tissue was ground in liquid nitrogen, lysed with NP‐40, and the supernatant was collected. The culture supernatant was also collected after centrifugation. Detection was performed according to the ELISA kit instructions, with results expressed in pg/mg prot.

### 2.6 Lactate and Pyruvate Detection

Lactate and pyruvate detection kits were purchased from Nanjing Jiancheng Bioengineering Institute. Lactate detection involved a standard curve method. Briefly, enzyme reserve solution and diluent were mixed at a 1:100 ratio, and the coloring solution was diluted to 6 mL. Standard samples were prepared, mixed with enzyme working solution and coloring agent, and incubated in a cell culture incubator for 10 min. Absorbance at 530 nm was measured to construct the standard curve. Brain tissue supernatant was prepared following the ELISA method to detect lactate levels. Pyruvate detection also used a standard curve method, with absorbance measured at 505 nm using a microplate reader to detect pyruvate levels in brain tissue supernatant.

### 2.7 Tissue Staining

Brain tissue was sectioned at 4 μm thickness using a Leica CM1500 cryostat and mounted on slides. The tissue slides were fixed with 40 g/L paraformaldehyde at room temperature for 15 min, washed three times with PBS for 5 min each, and blocked with 100 mL/L horse serum for 1 h. CD86 monoclonal antibody was applied and incubated overnight at 4°C. After washing the primary antibody with PBS three times for 5 min each, the secondary antibody was applied and incubated at room temperature in the dark for 2 h. The slides were then washed three times with PBS for 5 min each, stained with Hoechst dilution (1:5000) for 15 min, and mounted with 750 g/L glycerol. The mounted sections were stored at −20°C in the dark.

### 2.8 Western Blotting

Tissue homogenates were lysed with NP‐40 to extract proteins, and protein concentration was determined and adjusted using the BCA method. Protein electrophoresis was performed using precast gels. Protein samples were supplemented with 5× loading buffer to a volume of 20 μL, boiled for 8 min, and then electrophoresed at 80 V. The voltage was increased to 120 V for continued electrophoresis. The proteins were transferred to a membrane at a constant current of 300 mA for 0.5–2 h. The membrane was blocked with 5% skim milk for 2 h, incubated with primary antibodies diluted in TBST overnight on a shaker, and then incubated with horseradish peroxidase‐labeled goat anti‐rabbit secondary antibodies. After incubation, proteins were detected using chemiluminescence, and optical density was analyzed using Image Pro‐Plus 6.0 software.

### 2.9 Cell Model

Primary microglia were used, purchased from Procell Biotechnology Co. Ltd. Microglia were treated with 100 ng/mL PMA, and after adherent growth, they were used for experiments. After 24 h of PMA treatment, microglia were induced for M1 polarization with 100 ng/mL LPS and 20 ng/mL IFN‐γ for 24 h. Cells were divided into control, LPS + IFN‐γ, URI‐L, and URI‐H groups. The control group served as the control, whereas URI groups were pretreated with 5 mg/L and 10 mg/L uridine for 6 h before M1 polarization induction.

### 2.10 Flow Cytometry

After M1 polarization induction of microglia with LPS + IFN‐γ, all cells were collected, centrifuged at 3000 *g*, washed twice with cold PBS, and fixed with methanol. FITC‐F4/80, APC‐CD86, and PE‐CD11b monoclonal antibodies (10 μL each) were incubated in the dark for 30 min, washed twice with PBS, and resuspended in 50 μL of solution for detection. Results were expressed as a percentage.

### 2.11 Cell Fluorescence Staining

Microglia were stained for CD86 using the coverslip method. After induction with LPS + IFN‐γ, cells were washed three times with PBS, fixed with 4% formaldehyde at room temperature for 0.5 h, permeabilized with 0.2% Triton X‐100 for 5 min, and incubated overnight at 4°C with CD86 monoclonal antibody (1:400 dilution). After washing twice with PBS, fluorescent secondary antibodies were applied, and cells were mounted with 95% glycerol for observation under a fluorescence microscope.

### 2.12 Statistical Methods

All measurement data are expressed as (Mean ± SD). Statistical analysis was performed using SPSS 17.0 software. After homogeneity of variance testing, two independent sample *t*‐tests were used for comparisons between two groups; one‐way ANOVA was used for comparisons among three or more groups, followed by pairwise comparisons using the LSD method. All tests were two‐sided, with *p* < 0.05 considered statistically significant.

## 3 Results

### 3.1 Uridine Improves Cognitive Impairment and Microglial Polarization in AD Mice

**FIGURE 1:**
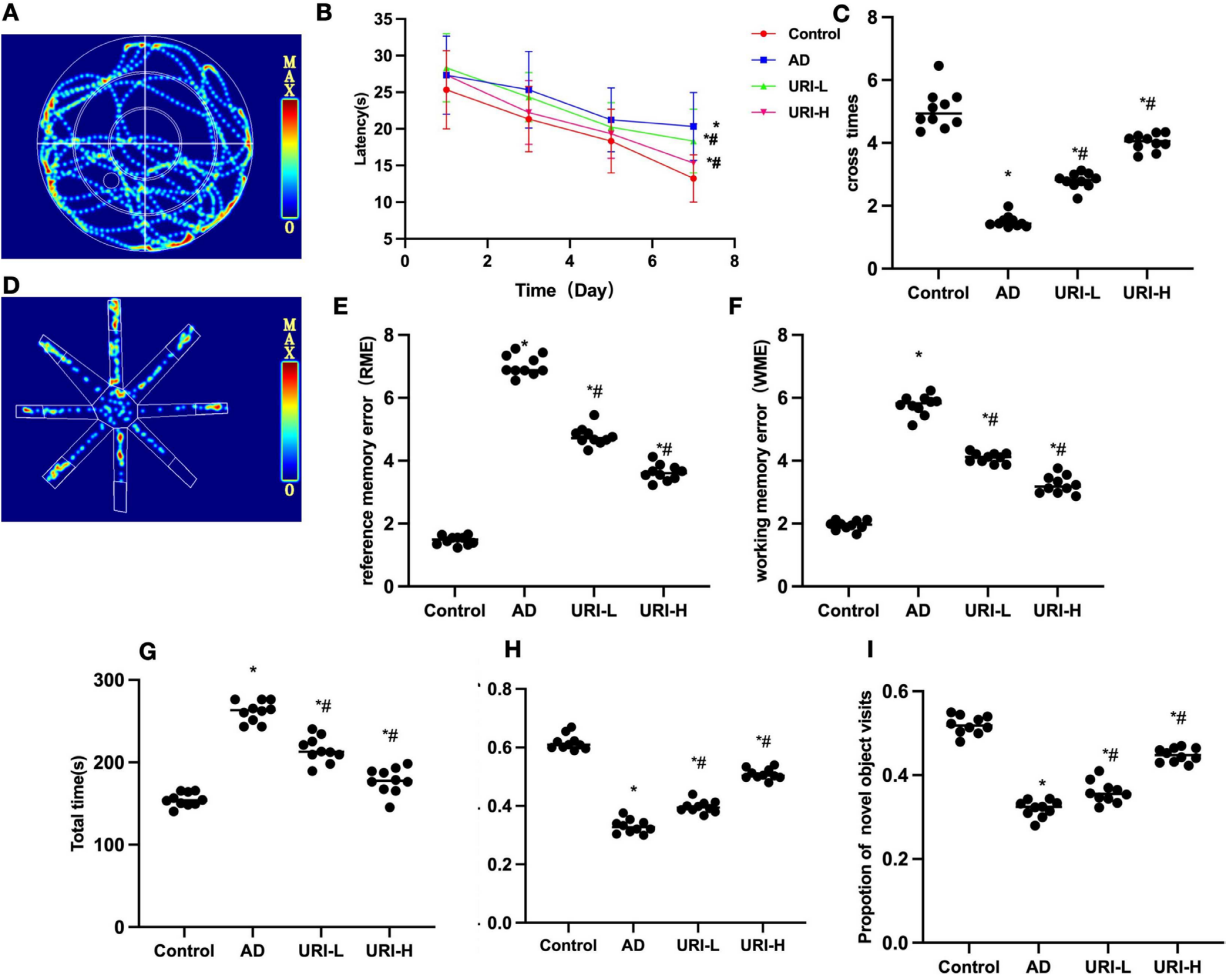
Effects of uridine on cognitive behavior in AD mice. (A–C): Morris water maze (*n* = 10). AD mice had significantly higher escape latency and lower platform crossings compared to control. URI reduced escape latency and increased platform crossings in a dose‐dependent manner. (D–G) Eight‐arm maze (*n* = 10). AD mice had significantly higher RME, WME, and total time compared to control. URI reduced these parameters significantly compared to AD, with a dose‐dependent effect. (H–I) Novel object recognition test (*n* = 10). AD mice had significantly lower touching time and frequency of the new object compared to Control. URI increased these values. *p < 0.05 compared to Control; #*p* < 0.05 compared to AD.

In AD mice, URI treatment improved cognitive impairment while inhibiting M1 cell polarization and reducing neuroinflammation levels. Morris maze results showed that AD mice had significantly higher escape latency and lower platform crossings compared to Control. URI reduced escape latency and increased platform crossings in a dose‐dependent manner (Figure 1A–C). Eight‐arm maze results indicated that AD mice had significantly higher RME, WME, and total time compared to Control. URI reduced these parameters significantly compared to AD, with a dose‐dependent effect (Figure 1D–G). In the novel object recognition test, AD mice had significantly lower touching time and frequency of the new object compared to Control. URI increased these values (Figure 1H,I).

**FIGURE 2:**
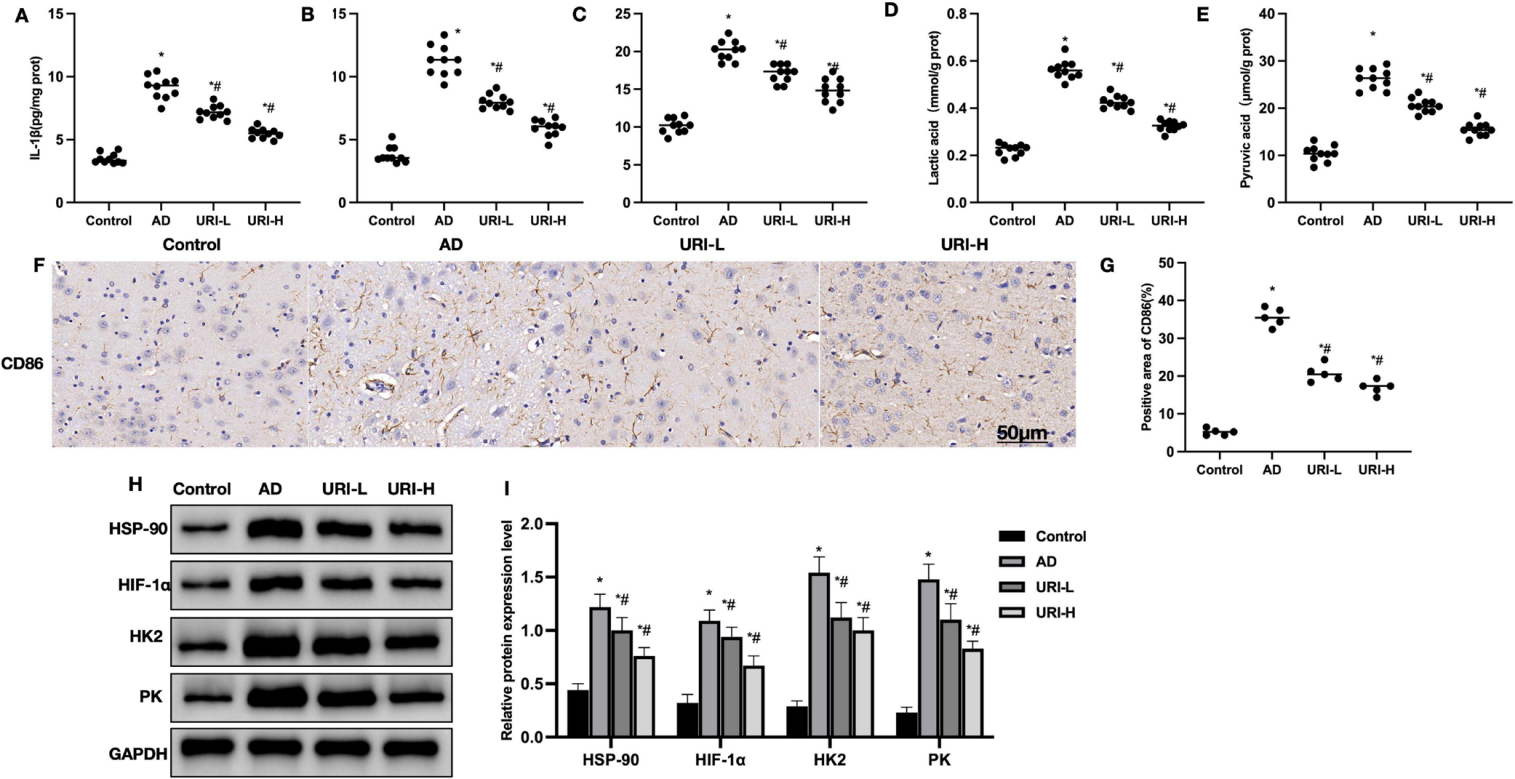
Effects of uridine on AD pathology. (A–C) ELISA (*n* = 10). IL‐1β, IL‐6, and TNF‐α levels were significantly higher in AD mice compared to Control. URI reduced these cytokine levels. (D–E) Lactate and pyruvate levels (*n* = 10). AD mice had higher levels of pyruvate and lactate compared to control, which were reduced by URI. (F–G) Tissue staining (*n* = 10). The proportion of CD86‐positive cells was higher in AD mice compared to control, and URI reduced these proportions. (H–I) Protein relative expression levels (*n* = 5). HSP90, HIF‐1α, and key glycolysis enzymes HK2 and PK were significantly higher in AD mice compared to Control. URI reduced these levels in a dose‐dependent manner. *p < 0.05 compared to control; #*p* < 0.05 compared to AD.

Detection of cortical inflammatory cytokines revealed that IL‐1β, IL‐6, and TNF‐α levels were significantly higher in AD mice compared to Control. URI reduced these cytokine levels (Figure 2A–C). Lactate and pyruvate detection showed that AD mice had higher levels of pyruvate and lactate compared to Control, which were reduced by URI (Figure 2D,E). Tissue staining results indicated that the proportion of CD86‐positive cells was higher in AD mice compared to Control, and URI reduced these proportions (Figure 2F,G). Protein relative expression levels showed that HSP90, HIF‐1α, and key glycolysis enzymes HK2 and PK were significantly higher in AD mice compared to Control. URI reduced these levels in a dose‐dependent manner Figure 2H,I.

### 3.2 Network Pharmacology Analysis Results

**FIGURE 3:**
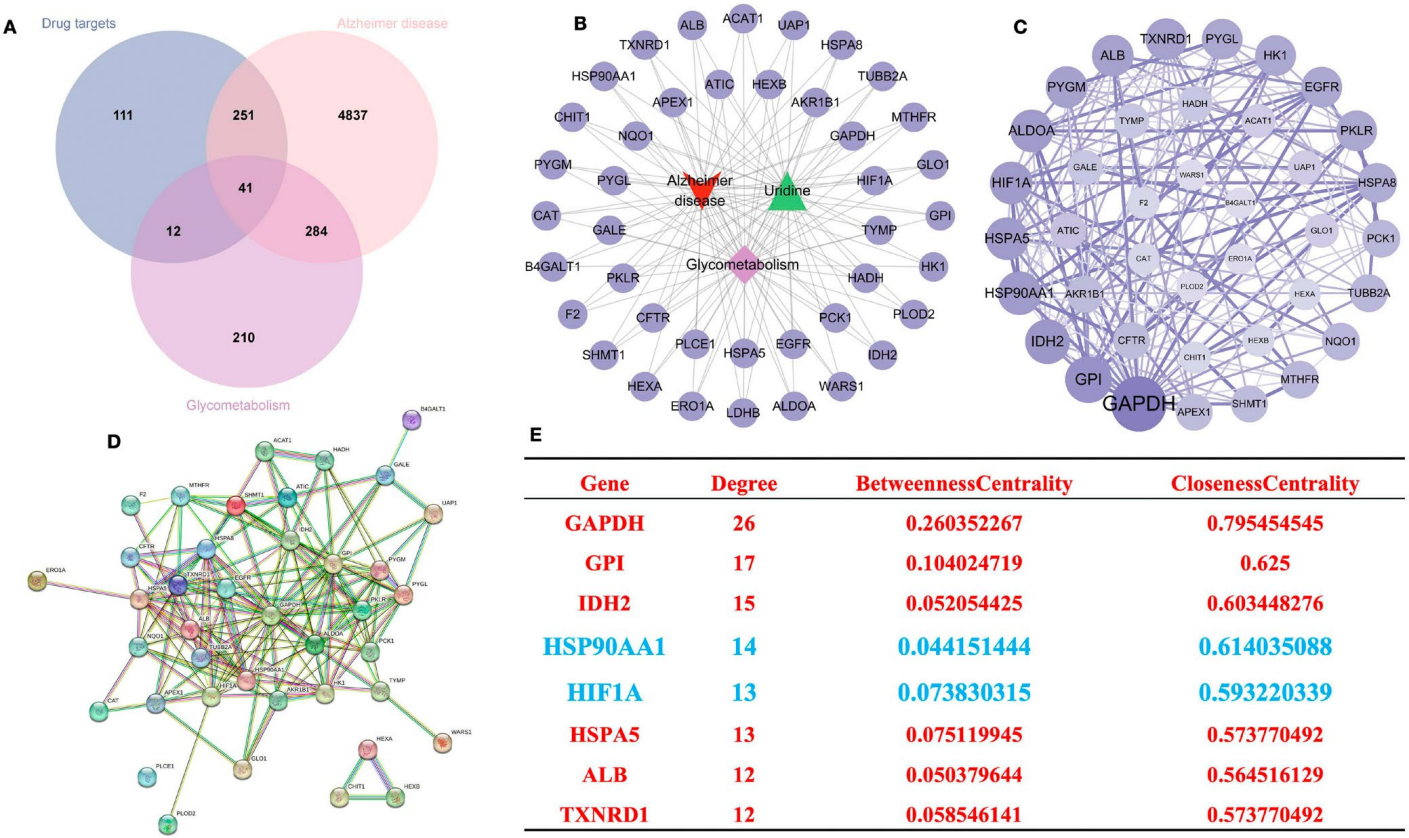
Network pharmacology analysis results of uridine. (A) Intersection of uridine‐AD‐glycolysis targets. (B) Visualization of intersection targets. (C, D) Protein–protein interaction (PPI) network. (E) Target ranking results.

A total of 415 uridine‐related targets were obtained from the Swiss Target Prediction, SEA, CTD, and Pharmmapper databases after removing duplicates. A total of 5413 and 547 Alzheimer's disease‐glycolysis‐related targets were retrieved from the GeneCards, CTD, OMIM, and KEGG databases. Intersection with uridine targets resulted in 41 potential targets related to the treatment of Alzheimer's disease‐glycolysis (Figure 3A). The network contained 44 nodes (including 1 active ingredient node, 41 target nodes, and 2 disease nodes) and 123 edges. Network Analyzer plugin was used to analyze the network's topological parameters: average adjacent nodes were 5.906, network heterogeneity was 5.591, network density was 0.130, and network centrality was 0.863 (Figure 3B). The 41 intersection targets were imported into the String database to clarify the interactions between component targets, resulting in 39 proteins and 154 interaction edges. The deeper the color, the higher the Degree value, indicating a more significant role of the protein in the network. The larger the node area, the more important the drug‐disease intersection and target protein interactions (PPI). The PPI information was imported into Cytoscape software for visualization analysis (Figure 3C,D). Screening criteria were based on Betweenness, Closeness, and Degree values, with targets above average considered core targets. The top 5 degree values were GAPDH (Degree = 26), GPI (Degree = 17), IDH2 (Degree = 15), HSP90AA1 (Degree = 14), and HIF1A (Degree = 13) (Figure 3E). These five targets are considered core targets playing a significant role in the development of Alzheimer's disease‐glycolysis treated by uridine.

**FIGURE 4:**
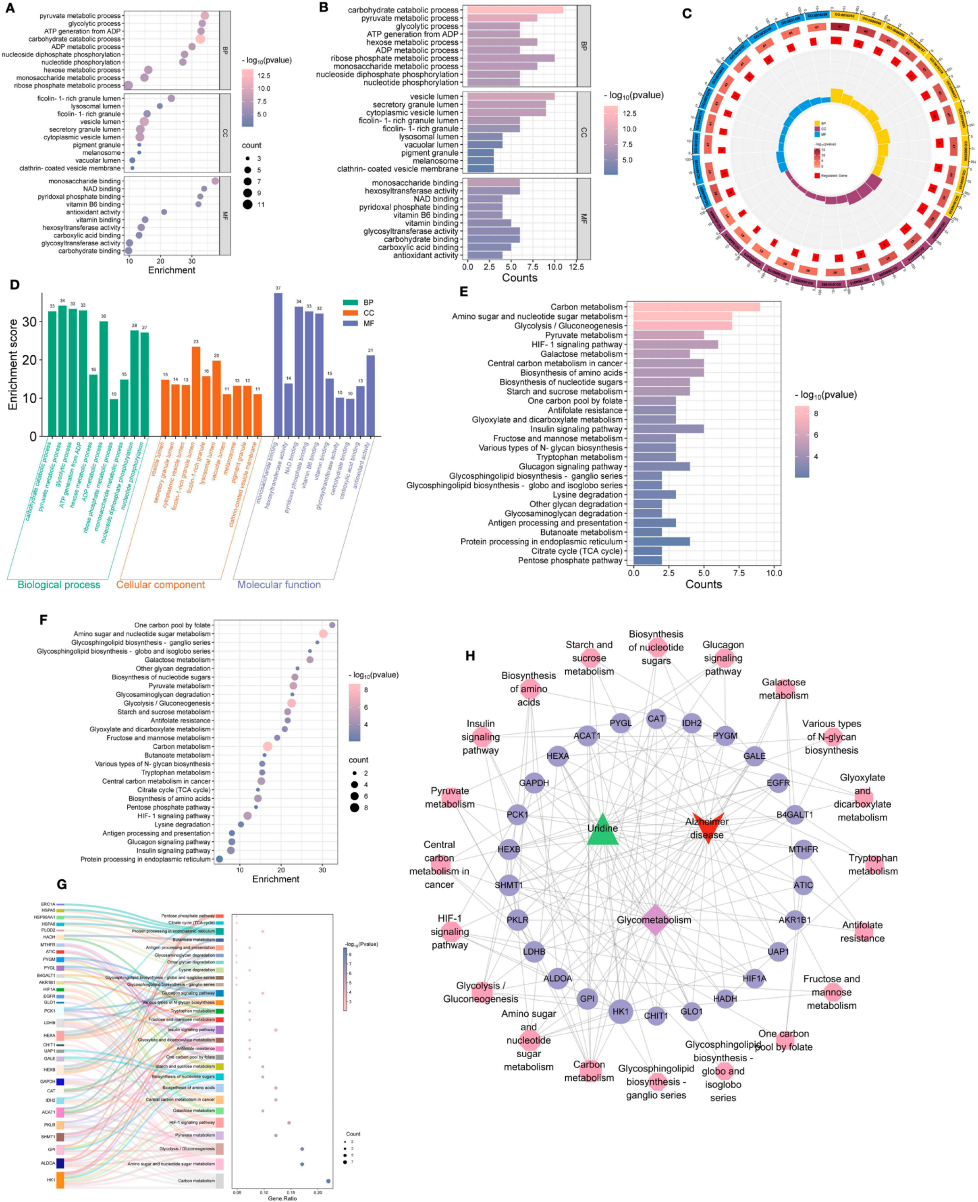
GO and KEGG analysis results. (A–E) GO analysis results. Potential targets are involved in biological functions such as carbohydrate metabolism, pyruvate metabolism, and glycolysis, all of which are closely related to the occurrence of Alzheimer's disease–glycolysis. (F, G): KEGG analysis results. A total of 28 signaling pathways were identified for uridine intervention in Alzheimer's disease–glycolysis. (H) Active ingredient‐target‐pathway network.

From KEGG enrichment analysis and GO enrichment analysis, the main biological processes can be located where target proteins are involved. R software was used for GO function enrichment analysis and KEGG pathway enrichment analysis to study the role of 189 intersection targets involved in Alzheimer's disease‐glycolysis in gene function and signaling pathways. The analysis showed that potential targets are involved in biological functions such as carbohydrate metabolism, pyruvate metabolism, and glycolysis, all of which are closely related to the occurrence of Alzheimer's disease‐glycolysis. This indicates that uridine's therapeutic effects on Alzheimer's disease‐glycolysis involve multiple biological processes (Figure 4A–E). Using the “Cluster Profiler” package in R software, KEGG pathway enrichment analysis was performed on 41 intersection targets of uridine for Alzheimer's disease‐glycolysis (Figure 4F,G). A total of 28 signaling pathways (*q* < 0.05) were identified for uridine intervention in Alzheimer's disease‐glycolysis. The enrichment results were visualized. In the “active ingredient‐target‐pathway” network, there were 49 nodes (including 1 active ingredient node, 26 target nodes, 20 signaling pathway nodes, and 2 disease nodes) and 165 edges. Network Analyzer plugin was used to analyze the network's topological parameters: average adjacent nodes were 6.735, network heterogeneity was 0.789, network density was 0.140, and network centrality was 0.418 (Figure 4H).

### 3.3 HSP90‐KO Inhibits the Effects of Uridine

**FIGURE 5:**
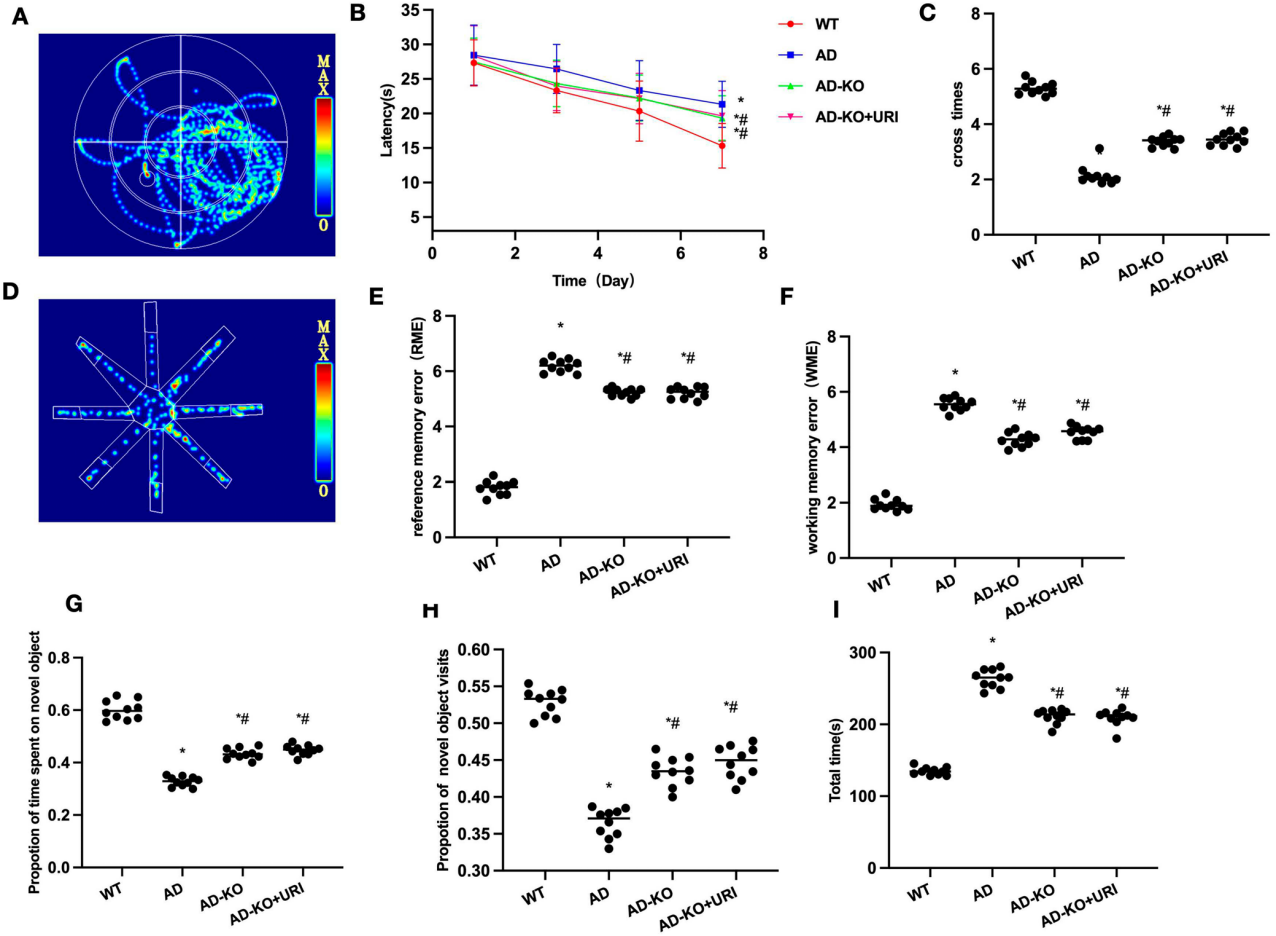
HSP90‐KO inhibits the effects of uridine. (A–C) Morris water maze (*n* = 10). Escape latency and platform crossings in AD‐KO and AD‐KO + URI mice were significantly different compared to AD, but not between the two groups. (D–G) Eight‐arm maze (*n* = 10). There were no significant differences between AD‐KO and AD‐KO + URI in RME, WME, and total time, but both were significantly lower than AD. (H, I) Novel object recognition test (*n* = 10). There were no significant differences between AD‐KO and AD‐KO + URI in touching time and frequency of the new object, but both were significantly lower than AD. *p < 0.05 compared to control; #*p* < 0.05 compared to AD.

We used AAV‐shRNA to silence HSP90 expression. Cognitive impairment in AD‐HSP90 mice was improved, and the proportion of M1 cells and microglial activation was inhibited. In AD‐KO mice, URI did not further improve cognitive impairment. Morris maze results showed that escape latency and platform crossings in AD‐KO and AD‐KO + URI mice were significantly different compared to AD, but not between the two groups (Figure 5A–C). Eight‐arm maze results indicated no significant differences between AD‐KO and AD‐KO + URI in RME, WME, and total time, but both were significantly lower than AD (Figure 5D–G). In the novel object recognition test, there were no significant differences between AD‐KO and AD‐KO + URI in touching time and frequency of the new object, but both were significantly lower than AD (Figure 5H,I).

**FIGURE 6:**
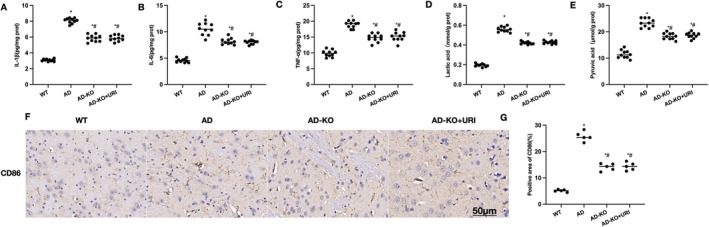
Effects of uridine on tissue pathology after HSP90‐KO. (A–C) ELISA (*n* = 10). There were no significant differences between AD‐KO and AD‐KO + URI in IL‐1β, IL‐6, and TNF‐α levels, but both were significantly lower than AD. (D, E) Lactate and pyruvate levels (*n* = 10). There were no significant differences between AD‐KO and AD‐KO + URI in pyruvate and lactate levels, which were lower than those in AD. (F, G) Tissue staining (*n* = 10). The proportion of CD86‐positive cells was higher in AD‐KO and AD‐KO + URI compared to AD, but there were no significant differences between the two groups. *p < 0.05 compared to control; #*p* < 0.05 compared to AD.

Inflammatory cytokine detection showed no significant differences between AD‐KO and AD‐KO + URI in IL‐1β, IL‐6, and TNF‐α levels, but both were significantly lower than AD (Figure 6A–C). Lactate and pyruvate detection revealed no significant differences between AD‐KO and AD‐KO + URI in pyruvate and lactate levels, which were lower than those in AD (Figure 6D,E). Tissue staining results indicated that the proportion of CD86‐positive cells was higher in AD‐KO and AD‐KO + URI compared to AD, but there were no significant differences between the two groups (Figure 6F,G).

### 3.4 Effects of Uridine on Microglial M1 Polarization

**FIGURE 7:**
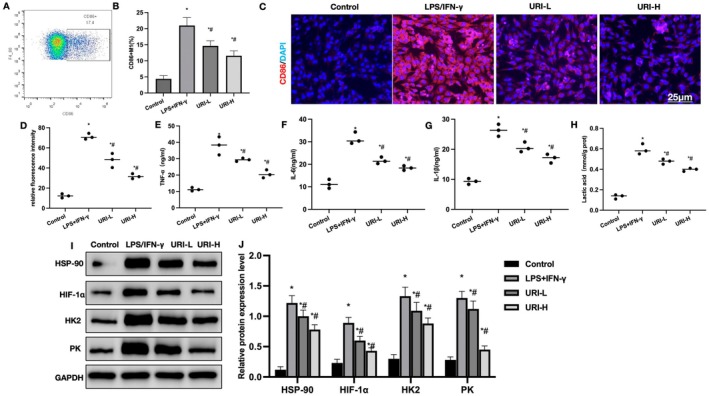
Uridine inhibits microglial M1 polarization. (A, B) FCM (*n* = 3). URI inhibited the polarization of CD86+ M1 cells, with a significant reduction in the CD86+ cell proportion compared to LPS + IFN‐γ in a dose‐dependent manner. (C, D) IF (*n* = 3). Strong CD86 positivity and higher fluorescence intensity in LPS + IFN‐γ compared to control. URI significantly weakened the fluorescence intensity, lower than LPS + IFN‐γ. (E–G) ELISA (*n* = 3). Higher inflammatory cytokine levels in LPS + IFN‐γ compared to control, which were inhibited by URI. (H) Lactate levels (*n* = 3). URI inhibited lactate levels, which were significantly lower than LPS + IFN‐γ. (I, J) Protein relative expression levels (*n* = 3). URI inhibited the expression of HSP90 and HIF‐1α in microglia, along with the levels of HK2 and PK. *p < 0.05 compared to control; #*p* < 0.05 compared to LPS + IFN‐γ.

In vitro cultured microglia were induced for M1 polarization with LPS + IFN‐γ. URI intervention inhibited M1 polarization and glycolysis in microglia. FCM results showed that URI inhibited the polarization of CD86+ M1 cells, with a significant reduction in CD86+ cell proportion compared to LPS + IFN‐γ in a dose‐dependent manner (Figure 7A,B). IF staining results indicated strong CD86 positivity and higher fluorescence intensity in LPS + IFN‐γ compared to Control. URI significantly weakened the fluorescence intensity, lower than LPS + IFN‐γ (Figure 7C,D). Inflammatory cytokine detection showed higher levels in LPS + IFN‐γ compared to Control, which were inhibited by URI (Figure 7E–G). URI also inhibited lactate levels, significantly lower than LPS + IFN‐γ (Figure 7H). Protein relative expression levels showed that URI inhibited the expression of HSP90 and HIF‐1α in microglia, along with the levels of HK2 and PK (Figure 7I,J).

### 3.5 Molecular Dynamics Analysis of Uridine and HSP90

**FIGURE 8:**
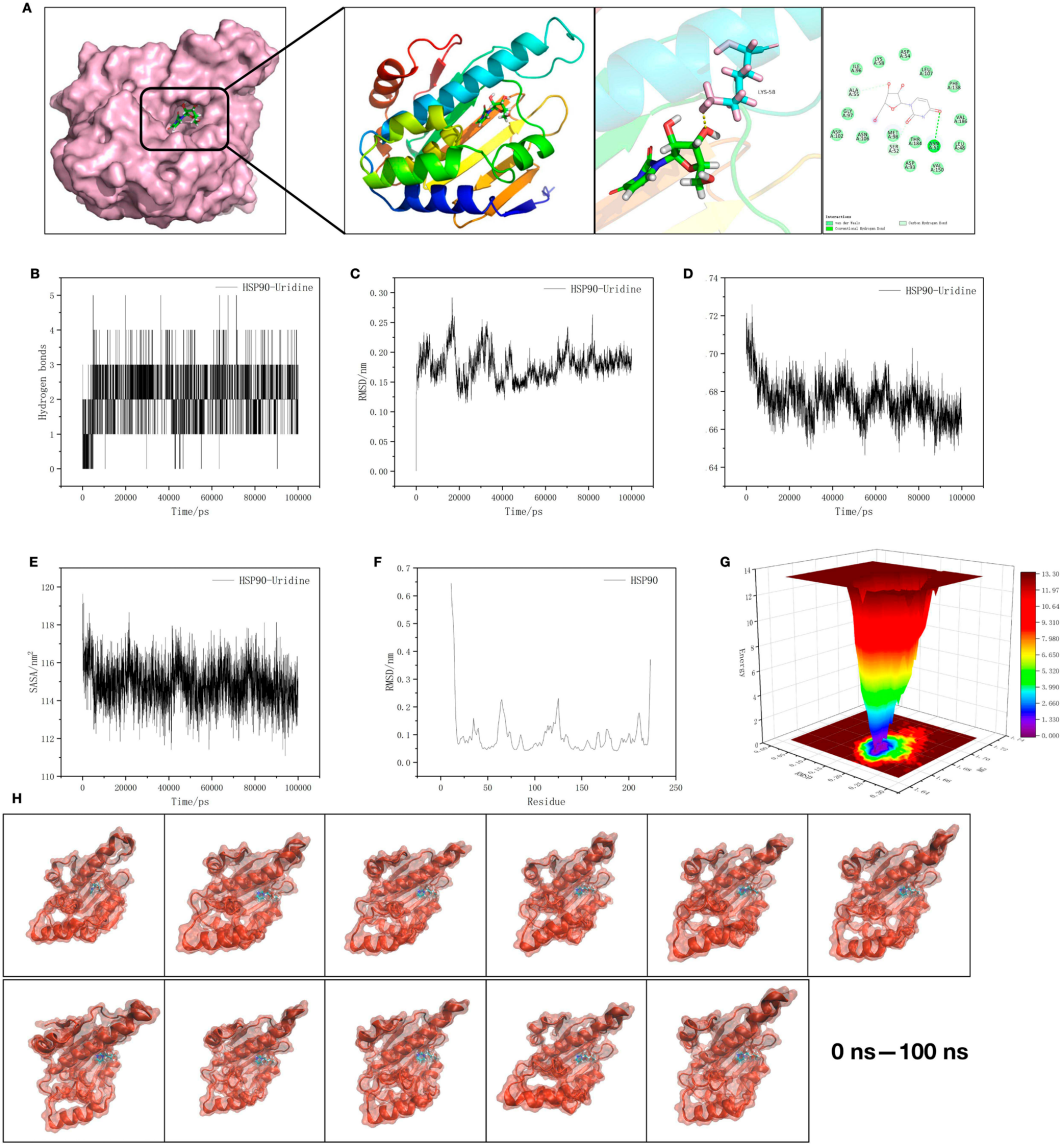
Molecular dynamics analysis of uridine and HSP90. (A) Docking results of uridine and HSP90. (B) Visualization of hydrogen bonds. (C–F) Molecular dynamics analysis. Stable binding in RMSD (0.15–0.25), RG (1.66–1.70), and SASA (113–117), with the lowest energy of HSP90 in RMSD. (G) Energy trap analysis results. (H) Visualization of binding over 0–100 ns.

Docking of uridine and HSP90 showed that URI binds to the cavity of HSP90, forming hydrogen bonds with ASN and THR (Figure 8A). The hydrogen bonds were visualized (Figure 8B). Molecular dynamics analysis showed stable binding in RMSD (0.15–0.25), RG (1.66–1.70), and SASA (113–117), with the lowest energy of HSP90 in RMSD (Figure 8C–F). The energy trap of URI‐HSP90 was visualized Figure 8G, and the binding was visualized over 0–100 ns Figure 8H.

## 4 Discussion

Microglia‐mediated neuroinflammation plays a significant role in the development of AD [8]. During microglial infiltration and M1 polarization, various inflammatory cytokines are expressed and released, inducing the production of reactive oxygen species (ROS) and activating the NF‐κB signaling pathway [16, 17, 18]. Recent studies have also shown that glycolysis is one of the primary energy pathways for M1 polarization, with glycolysis playing a crucial role in the inflammatory activation of M1 microglia. Similarly, blocking glycolysis can inhibit M1 polarization. HIF‐1α, as an essential hypoxia response factor [19], has been reported to mediate glycolysis, promoting M1 polarization in macrophages. Blocking HIF‐1α has become a primary means of inhibiting cellular glycolysis and M1 polarization [11]. HIF‐1α expression is regulated by several heat shock proteins. Research has shown that HSP90 stabilizes HIF‐1α, promoting its expression and activation while increasing glycolysis levels [20]. Additionally, HSP90 can enhance the expression of glycolytic enzyme PKM2, working synergistically with HIF‐1α. Current research has proven that the HSP90‐HIF‐1α signaling pathway is a major promoter of glycolysis [21, 22].

Uridine, a nucleoside small molecule abundant in deer antler, has been found to delay stem cell aging and promote tissue regeneration and repair, as well as regulate biological rhythms. Although uridine regulates neuronal metabolism and function, its targets have not been clearly identified. In our study, after administering uridine from deer antler, we observed significant improvements in cognitive impairment in AD mice. Memory capacity, novel object recognition ability, and motor skills were enhanced. Uridine also reduced tissue inflammatory cytokine levels. The M1 polarization state of microglia is one of the main pathological manifestations of neuroinflammation in AD. In addition to reducing inflammatory cytokines, uridine inhibited M1 polarization in microglia, with a significant decrease in the proportion of CD86+ cells, a membrane protein marker of M1 cells [12]. Uridine also reduced the proportion of IBA‐1+ positive cells, a marker of microglial activation. Further detection revealed that uridine decreased the expression of HSP90 and HIF‐1α, along with glycolytic enzyme levels [23]. Studies have shown that HIF‐1α promotes glycolysis through genes such as HK2 and PFK1 [24]. When HIF‐1α is absent, cells exhibit reduced glycolytic rates and energy metabolism levels [25]. Our network pharmacology analysis further revealed multiple intersection targets in the uridine‐AD‐glycolysis pathway, with HSP90 having a high score. Uridine was also highly correlated with metabolic pathways, supporting our experimental findings.

When we silenced HSP90 using HSP90‐shRNA, the effects of uridine were inhibited. Similarly, HSP90‐KO improved cognitive impairment in mice. When uridine was administered in HSP90‐KO mice, it did not further enhance cognitive improvement or significantly affect the proportion of CD86+ and IBA‐1+ cells. The proportion of CD86+ and IBA‐1+ cells in HSP90‐KO mice was lower than that in AD mice, indicating that HSP90 is one of the crucial factors promoting M1 polarization in microglia in AD. Our in vitro experiments also verified the effects of uridine. LPS + IFN‐γ is one of the methods to induce M1 polarization [26]. LPS + IFN‐γ promoted M1 polarization in primary microglia, with a significant increase in the proportion of CD86+ cells. Uridine inhibited this process, reducing the proportion of M1 cells and inflammatory cytokine expression while also affecting the HSP90‐HIF‐1α signaling pathway. The in vitro and in vivo experimental results were consistent.

## 5 Conclusion

Our study investigated the effects of uridine, an active component in deer antler, on cognitive impairment in AD. The results showed that uridine significantly improved cognitive and behavioral deficits in AD mice. Its mechanism is related to inhibiting microglial glycolysis and M1 polarization, reducing neuroinflammation. Our research provides new insights and references for the pharmacological effects of deer antler. However, how to apply URI to clinical treatment needs further research, which is also the direction for future expansion of this study.

## Conflicts of Interest

The authors declare no conflicts of interest.

## Data Availability

The authors have nothing to report.
